# Morphological Characterization and Transcriptional Regulation of Corolla Closure in *Ipomoea purpurea*

**DOI:** 10.3389/fpls.2021.697764

**Published:** 2021-09-07

**Authors:** Peipei Zhang, Mingyue Sun, Xiaoqiong Wang, Runjiu Guo, Yuchu Sun, Mengyuan Gui, Jingyuan Li, Taixia Wang, Liang Zhang

**Affiliations:** ^1^College of Life Science, Henan Normal University, Xinxiang, China; ^2^Engineering Technology Research Center of Nursing and Utilisation of Genuine Chinese Crude Drugs in Henan Province, Xinxiang, China; ^3^Key Laboratory of Plant Resources, Institute of Botany, Chinese Academy of Sciences (CAS), Beijing, China

**Keywords:** corolla closure, bulliform cells, acuminate veins, transcriptomes analysis, differentially expressed genes

## Abstract

Corolla closure protects pollen from high-temperature stress during pollen germination and fertilization in the ornamental plant morning glory (*Ipomoea purpurea*). However, the morphological nature of this process and the molecular events underpinning it remain largely unclear. Here, we examined the cellular and gene expression changes that occur during corolla closure in the *I*. *purpurea*. We divided the corolla closure process into eight stages (S0–S7) based on corolla morphology. During flower opening, bulliform cells appear papillate, with pigments in the adaxial epidermis of the corolla. These cells have distinct morphology from the smaller, flat cells in the abaxial epidermis in the corolla limb and intermediate of the corolla. During corolla closure, the bulliform cells of the adaxial epidermis severely collapse compared to cells on the abaxial side. Analysis of transparent tissue and cross sections revealed that acuminate veins in the corolla are composed of spiral vessels that begin to curve during corolla closure. When the acuminate veins were compromised, the corolla failed to close normally. We performed transcriptome analysis to obtain a time-course profile of gene expression during the process from the open corolla stage (S0) to semi-closure (S3). Genes that were upregulated from S0 to S1 were enriched in the polysaccharide degradation pathway, which positively regulates cell wall reorganization. Senescence-related transcription factor genes were expressed beginning at S1, leading to the activation of downstream autophagy-related genes at S2. Genes associated with peroxisomes and ubiquitin-mediated proteolysis were upregulated at S3 to enhance reactive oxygen species scavenging and protein degradation. Therefore, bulliform cells and acuminate veins play essential roles in corolla closure. Our findings provide a global understanding of the gene regulatory processes that occur during corolla closure in *I. purpurea*.

## Introduction

Flowers are hugely diverse, with variations in characteristic features such as shape, size, color, arrangement, and flowering time. Morphological changes in flowers attract insects and allow plant to adapt to the environment to facilitate breeding mechanisms. Many plant species are capable of moving some portions of flower structure, such as petals/corolla, pistils, and stamens, in response to internal and/or external factors (Ruan and da Silva, 2011; Liu et al., 2017). Corolla movement, one of the most extensively studied petals movement in flowers, is important for plant productivity and ornamental value (Bynum and Smith, 2001; Frund et al., 2011; van Doorn and Kamdee, 2014; Liu et al., 2017). During anthesis, flowers of some plants, such as *Arabidopsis thaliana* and rose, remain open until the petals wither, whereas others flowers close during this process. The permanent closure of the corolla is often accompanied by senescence in plants such as *Ipomoea purpurea* (Shibuya et al., 2014; Gui et al., 2016; Liu et al., 2020) and *Mirabilis jalapa* (Hu et al., 2013). The temporal closure and repeated opening of petals in plants such as *Nymphaea colorata* (Ke et al., 2018), *Eustoma grandiflorum* (Bai and Kawabata, 2015), and *Gentiana algida* (Bynum and Smith, 2001) are generally regulated by environment or rhythmic changes.

The complex mechanism of flower opening and closure has attracted extensive attention. From an evolutionary perspective, the closure of flowers may occur as a strategy to optimize reproductive success. Flower closure can be advantageous because it helps to provide an optimum environment for successful pollination, fertilization, and fruit set (van Doorn and Kamdee, 2014). For example, floral closure benefits reproduction in *Magnolia denudate* and *Crocus discolor* by protecting anther development and pollen viability against cold in early spring (Liu et al., 2017; Prokop et al., 2019). *Gentiana algida* flowers close fully to prevent pollen loss during thunderstorms (Bynum and Smith, 2001). We previously study that the closed corollas of morning glory (*Ipomoea purpurea*) form a bell shape that maintains a lower temperature compared to the external environment to ensure complete pollen germination and fertilization (Liu et al., 2020). Corolla closure also contributes to delayed selfing in *Kosteletzkya virginica* (Ruan et al., 2005).

Various types of floral movements are based on the reversible expansion and contraction of cells due to changes in osmotic pressure or differential elongation growth. Four types of physiological processes are known to affect flower movement: carbohydrate metabolism, cell wall expansion, water uptake, and hormonal regulation (van Doorn and Van Meeteren, 2003). Before flower opening, the levels of osmotic solutes increase via processes such as the conversion of polysaccharides to monosaccharides. In *Chrysanthemum* petals, starch and fructose are degraded during petal expansion (Trusty and Miller, 1991). In “Mitchell” *Petunia* variety, knockdown of genes encoding cell-wall-associated β-galactosidases, which determine galactan levels in cell wall polysaccharides in petals, severely reduces the angle of flower opening due to the disruption of petal integrity (O’Donoghue et al., 2017). Water uptake is followed by increased osmotic pressure. For example, *Silene saxifraga* petals are closed during the day due to net water loss and reopen at night due to refilling of the cells with water (Halket, 1931). Endogenous hormones such as ethylene and auxin have been shown to regulate corolla opening and closure. In *Nymphaeales* (also called waterlily), the adaxial cells of the intermediate segment of the petal are highly flexible following circadian cell expansion due to auxin stimuli (Ke et al., 2018). Rapid flower closure has been observed in the *Convolvulaceae* (to which *I. purpurea* belongs), as well as the *Portulacacea*e, after ethylene treatment (Van Doorn, 2002). Finally, mechanical stimulation and pollination can also induce flower closure (Frund et al., 2011; Tagawa et al., 2018). The underlying physiology of flower movement has been the focus of much recent research, but the process of flowers closure remains incompletely understood.

*Ipomoea purpurea* is an important ornamental plant with a unique flower shape, extraordinary color, and long flowering period (June to October). Its flowers have a funnel-shaped corolla that opens in the morning and generally assumes a bell shape due to corolla closure later on the same day (McCallum and Chang, 2016). Therefore, *I. purpurea* is an excellent model plant for studying the mechanisms of flower closure. Previous studies showed that the processes of flower opening are distinctly different from that of flower closing in *Ipomoea tricolor* (Phillips and Kende, 1980). During the stage of flower closing in *Ipomoea tricolor*, asymmetric turgor changes in the two sides of epidermal cells could affect the rolling up response of rib in corolla (Hanson and Kende, 1975; Phillips and Kende, 1980). In addition, the corolla cells of *Ipomoea* at the closure stage exhibit features of senescence, such as organelle degradation and membrane rupture (Phillips and Kende, 1980; Gui et al., 2016), indicating that corolla closure is closely related to process of cell senescence. Although the ecological roles and structural changes of flower closure in *I. purpurea* are reported, the morphological nature and molecular basis for corolla closure remain largely unclear.

In this study, we investigated the morphological and molecular basis of corolla closure in *I. purpurea*. Using scanning electron microscopy (SEM), we observed that the adaxial corolla epidermis contains papillary-shaped bulliform cells that break during the process of corolla closure, in contrast to the flat cells in the abaxial epidermis. Analysis of transparent tissue and cross-sections revealed that acuminate corolla veins comprising vessel structures play important roles in corolla closure. Furthermore, transcriptome analysis at different stages of corolla closure showed that carbohydrate metabolism, autophagy, and hormone signaling are coordinately involved regulating corolla closure. Analysis of gene expression patterns uncovered a detailed chronology of the transcriptional and functional changes that occur during corolla closure. Our results provide insights into the mechanisms that control corolla closure in *I. purpurea* and lay the foundation for the investigation of flower permanent closure.

## Materials and Methods

### Plant Materials and Growth Conditions

*Ipomoea purpurea* seeds were sown in soil in April and cultivated in a natural environment at the Ornamental Center of Henan Normal University, Xinxiang, China (35°19′N, 113°54′E). *I. purpurea* flowers from June until October. The flowers of *I*. *purpurea* open at dawn and close by late morning on the same day.

### Dynamic Observation of Corolla Closure

The closure of *I. purpurea* flowers was captured by photographing the flowers horizontally using a Canon EOS5D digital camera (Canon Inc., Tokyo, Japan). For movie scanning, images of flower closure were taken with a Canon EOS5D camera once per minute, and Premier Pro CS6 software (Adobe Systems Incorporated, United States) was used to generate a video.

### Microscopy

For scanning electron microscope (SEM), *I*. *purpurea* corollas at different stages of closure were fixed in 2.5% glutaraldehyde (pH 7.2), dehydrated in a graded ethanol series (30, 50, 70, 80, 90, 100, and 100% EtOH for 15–20 min each time), and transferred into an isoamyl acetate-EtOH (v/v) series (25, 50, 75, 100, and 100% for 20 min each time). Samples critically dried with a CO_2_ critical-point dryer (Leica EM CPD300, German). After being sputter-coated with gold, the dried petals were examined using a scanning electron microscope (Hitachi TM3030 plus, Japan) with 15 kV voltage and 11–13 mm working distance.

### Tissue Transparency and Preparation of Semi-Thin Sections

All samples were subjected to a modified tissue clearing method. Each corolla was made transparent via overnight incubation in a 10% NaOH solution and then stained with 1% sarfanine (70% alcohol) for 24–36 h. The samples were washed in 70% alcohol and observed under an illumination microscope (MVX10, Olympus, Japan) or photographed with a digital camera.

Samples were fixed in 0.1 mol/L phosphate buffer (pH 7.2) containing 2.5% glutaraldehyde, dehydrated in an ethanol series, infiltrated and embedded in EPON 812, and polymerized at 60°C for 24 h. Serial sections (2 μm) were cut with a Reichert-Jung ultramicrotome (Vienna, Austria) and stained with toluidine blue O. The sections were photographed under a light microscope (MVX10, Olympus, Japan).

### Analysis of Vessel Structures

Open and closed of *I. purpurea* corollas were collected and the veins segregated overnight in segregation solution [10% chromic acid:10% nitric acid = 1:1 (by vol.)]. The samples were washed three or four times with distilled water and preserved in 75% ethyl alcohol. Some samples were placed in a fresh tube, stained with 1% safranine for 5 h, and observed under a light microscope (MVX10, Olympus, Japan). The remaining samples were mounted on coverslips and completely dried at 37°C. After being sputter-coated with gold, the dried veins were examined under a scanning electron microscope (JSM-7800F, JEOL, Japan).

### Transcriptome Sequencing (RNA-Seq) and Data Processing

Corollas at four different stages, each with three biological replicates, were collected and ground into a powder in liquid nitrogen for RNA extraction. The integrity and concentration of the RNA were determined using an Agilent 2100 Bioanalyzer (RNA integrity number, RIN>6.0). Twelve independent libraries were constructed and analyzed on the Illumina HiSeq X platform at BGI Genomics Co., Ltd. (Shenzhen, China). The quality of all 12 transcriptional profiles was reflected by the clean read ratios (93.47–95.76%). A gene was regarded as being preferentially expressed if the FPKM (expected number of Fragments Per Kilobase of transcript sequence per Millions base pairs sequenced) at a specific point was at least 1 (FPKM ≥ 1.0). The clean read data were deposited in the National Genomics Data Center (NGDC) under the BioProject accession number PRJCA004887^1^.

### Clustering and KEGG Pathway Analysis of RNA-Seq Data

Before clustering, genes that were minimally expressed throughout the time course (as assessed by genes with a maximum expression level <1) were filtered out. Genes in specific clusters were chosen based on a |log_2_ (fold change)| ≥2 by comparing different stages (such as S1 vs. S0). The data for all remaining genes were Z-normalized using the standardize function in *Mfuzz software* with the number of clusters set to 6. Genes with membership scores >0.8 were considered to be part of a cluster. Pathway analysis of the DEGs in the different clusters was performed using KEGG^2^. Significantly enriched KEGG pathways (corrected *p*-value < 0.05) were identified based on a hypergeometric test.

### Quantitative Real-Time PCR

First-strand cDNA was synthesized from 1 μg of total RNA using a PrimeScript RT reagent Kit with gDNA Eraser (Takara Bio. Inc., Dalian, China). Diluted cDNA was used as a template in a 10 μl reactions mixture containing 20 ng of cDNA, 100 nM forward and reverse primers, and 5 μl TB Green Premix Ex Taq (Takara). Real-time quantitative PCR was conducted in Light Cycler^®^ 96 (Roche, Basle, Switzerland) following the manufacturer’s instructions. Three biological and two technical replicates were performed in each experiment. Parallel reactions were performed to amplify the *Actin* gene and used to normalize the amount of template. All primers and their sequences are listed in Supplementary Table 4. The primer specificity was validated by examining their melting profiles, showing a single product at a specific melting temperature. All PCR efficiencies were above 95%.

### Statistical Analysis

All data from three biological replicates were subjected to analysis of variances (ANOVA) according to the completely randomized design model using SPSS Statistics 17.0 (SPSS Inc., Chicago, United States). Statistical differences between the means of the plant lines and treatments were evaluated by Duncan’s test at the 0.05 probability level.

## Results

### The Morphological Features of Corolla Closure

**FIGURE 1:**
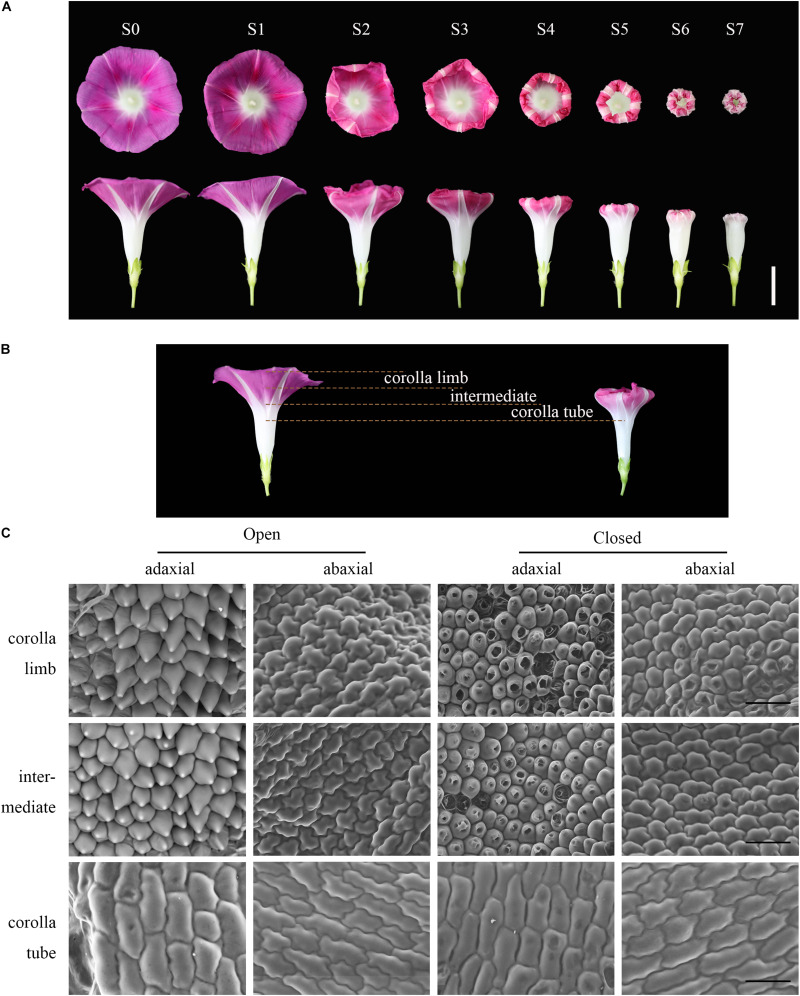
The process and micromorphology of corolla closure. **(A)** The process of corolla closure was captured with a camera. S0–S1: full bloom stage; S2–S3: early corolla closure stage; S4–S5: late corolla closure stage; S6–S7: full corolla closure stage. The upper images were taken at a high angle and the lower images were taken from the side. Scale bar = 2 cm. **(B)** Longitudinal section of a corolla showing that several corolla layers were all responsive to movement. Yellow dotted lines mark the corolla limb, intermediate and corolla tube segments of the corolla. The intermediate corresponds to the junction between the corolla limb and tube. **(C)** Cell morphology of the adaxial and abaxial epidermis at different stages was tracked by SEM. Open: S1; Closed: S3. Scale bar = 50 μm.

*Ipomoea purpurea* produces a sympetalous flower in which the upper part is funnel-shaped with a pink corolla and the lower part is an uncolored corolla tube. To explore floral movement of corolla closure, we recorded morphological changes with a camera (Figure 1A and Supplementary Movie 1). We divided the process of flowering (from full bloom to full closure) into eight stages (Figure 1A). During the first stage (S0), the corolla was in full bloom and trumpet-shaped, with the stamens and pistil exposed. The corolla can remain open for periods (S1), ranging from 4 to more than 10 h in the summer or early autumn. The midrib initially curled inward at S2 and the corolla began to gradually shrink from the edges and fold inward from S3 to S7. At S3 and S4, the corolla gradually folded inward by 1/3 and 2/3, respectively, until S5. The corolla then shrank inward and the midrib deformed during S6 and S7. During corolla closure, the flower diameter and height began to decrease, ultimately reaching the diameter of the cylindrical part of the lower corolla at S7. Although the corolla tube (white part) remained upright, the entire corolla assumed a bell shape and completely enclosed the stamens and pistil (Figure 1A).

To explore which part of the flower is responsible for this closure, we removed different parts of the flower. Interestingly, when 1/3 or 2/3 of the corolla was removed the corolla still folded inward (Supplementary Figures 1B,C). However, when the full corolla was removed, the flower no longer curled inward and completely failed to close (Supplementary Figure 1D). Previous studies have demonstrated the roles of corolla closure in pollination and fertilization (Liu et al., 2020). Here we showed that the corolla could still close when the stamens and pistil were artificially removed, indicating that pollination is not the only factor involved in corolla closure (Supplementary Figure 1A).

### The Cellular Basis of Corolla Closure

To investigate the cellular basis of corolla closure, we analyzed cell morphology in corolla limb, corolla tube, and the junction between the corolla limb and tube (intermediate) of the sympetalous corolla by SEM (Figure 1B). During flower opening, the cells in the adaxial side of the corolla limb presented papillae shape (bulliform cells), whereas the cells in the abaxial side were irregular and flat. The cell morphology in the intermediate part of the corolla was similar to that of the corolla limb. In contrast, long, narrow cells were observed on both the adaxial and abaxial sides at the corolla tube (Figure 1C). To further verify these results, we observed cell morphology in different segments of the corolla generated by freehand dissection (Supplementary Figure 2). At the corolla limb, the adaxial epidermal cells were large, triangular, and pink, suggesting that the color of the corolla is mainly derived from the large amount of pigments in these cells. In contrast, the abaxial epidermal cells were small, square, and tightly arranged, without a particular color (Supplementary Figure 2A). The morphology of the epidermal cells in the intermediate and corolla limb was similar (Supplementary Figure 2B). At the corolla tube, the cells of the adaxial and abaxial epidermis were square or rectangular and tightly arranged (Supplementary Figure 2C).

Water loss is thought to cause cellular collapse, which is likely to contribute to corolla closure (Yamada et al., 2006; Ke et al., 2018). Therefore, we examined the micromorphological features of corolla cells at S3 (i.e., semi-closure of the corolla). The epidermal cells on the corolla limb and intermediate of the corolla in the abaxial side showed obvious shrinkage. Most of the cells began to disintegrate and developed a cavity (Figure 1C, right). This cellular shrinkage was more pronounced in the abaxial side than in the adaxial side. However, the morphology of the corolla tube epidermal cells remained relatively intact without obvious shrinkage or collapse.

### Morphological Changes in Corolla Veins During Corolla Closure

**FIGURE 2:**
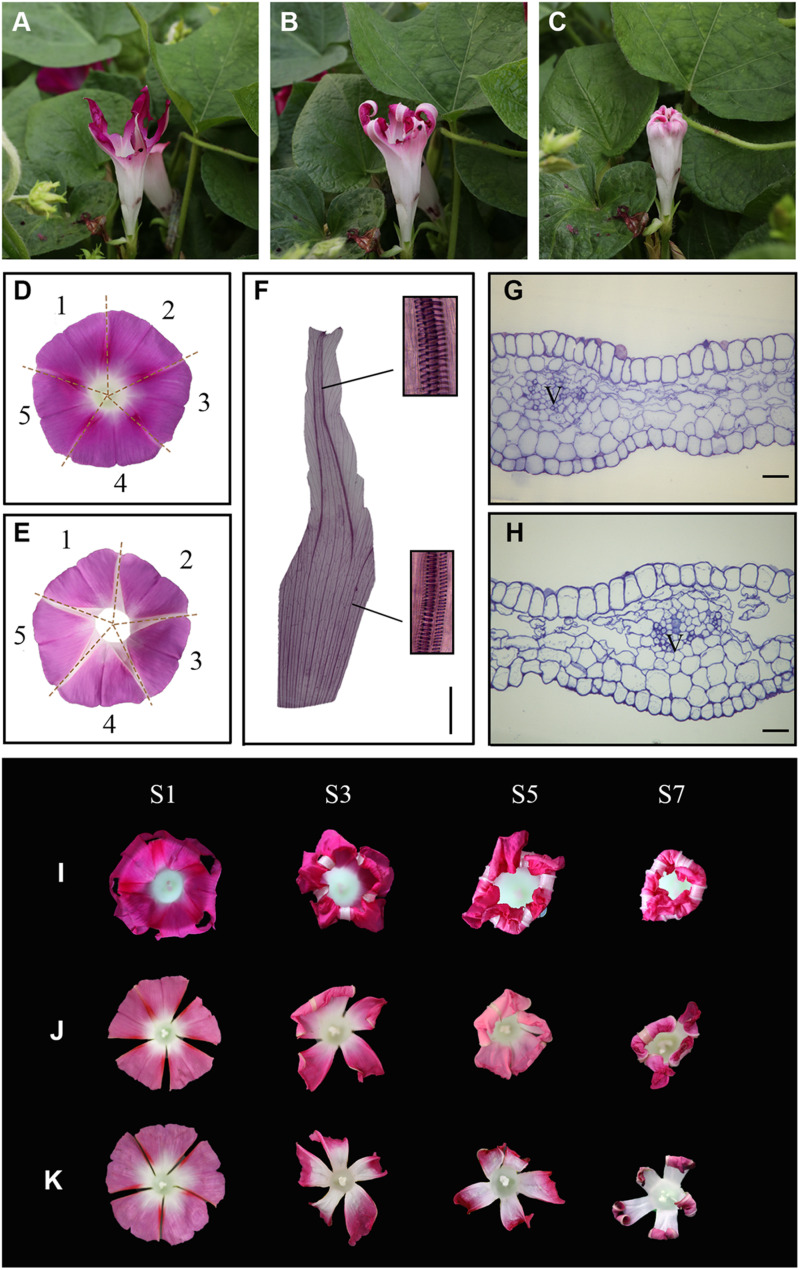
The acuminate veins maintain a trumpet shape to ensure corolla closure. **(A–C)** An insect-eaten corolla remains closed under natural conditions. **(D,E)** The white acuminate structures are arranged in the shape of a pentagram, and the sympetalous corolla consists of five parts. **(D,E)** show the adaxial and abaxial sides, respectively. **(F)** Structural observation of acuminate veins in transparent tissue. Magnified views of spiral vessels are shown in black boxes. Scale bar = 0.5 cm. **(G,H)** Transverse semi-thin sections of acuminate veins during the corolla opening **(G)** and closure **(H)** stages. V = vascular bundle. Scale bars = 50 μm. **(I–K)** The process of corolla closure was observed after the artificial destruction of acuminate veins. **(I)** 1/3 of the acuminate vein structure was removed. **(J,K)** Four or five acuminate veins were artificially destructed, respectively.

Interestingly, when whole corolla except for the white acuminate rib structures was eaten by insects under natural conditions, these structures gradually folded inward and closed into a bell shape (Figures 2A–C). These observations suggest that the acuminate rib structures play an important role in corolla closure. We therefore analyzed the acuminate rib structures in more detail by examining transparent tissue and transverse sections of tissues. The flower of *I*. *purpurea* contains a sympetalous corolla (Figures 2D,E) and acuminate rib structures arranged in the shape of a pentagram. Analysis of transparent tissue samples revealed that the acuminate rib structures are veins that comprise the spiral vessels (Figure 2F). The vessels run parallel in a vertical direction at the tube of the corolla, whereas the adjacent vessels at the limb of the corolla gradually close (Figure 2F). These vessel structures were also observed by transverse section (Figures 2G,H).

To confirm the roles of acuminate veins in corolla closure, we analyzed the response of the corolla to the artificial destruction of veins. We removed one-third of the acuminate veins of a flower, leaving the corolla loosely hanging without support (Figure 2I, stage S1). Surprisingly, the incomplete corolla folded inward along with the movement of the veins and ultimately closed into a bell shape (Figure 2I, stage S7). We also cut off four or five acuminate veins from corolla, resulting in a polypetalous corolla (Figures 2J,K). The rest of the corolla failed to fold inward when all acuminate veins were removed, but did fold when one acuminate vein remained intact, suggesting that acuminate veins play an important role in corolla closure in addition to supporting corolla structure.

**FIGURE 3:**
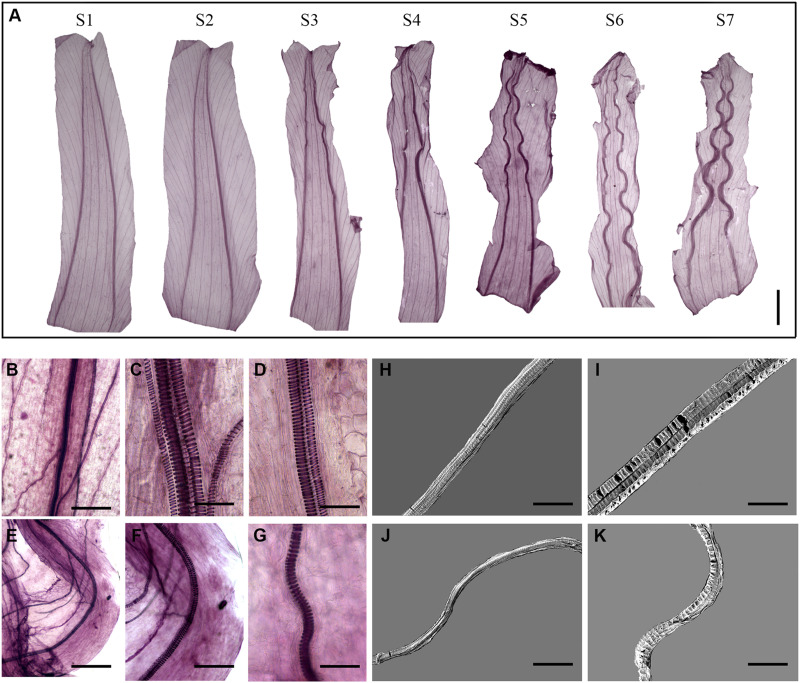
Corolla closure is accompanied by dynamic changes in acuminate vein structure. **(A)** Tissue transparency showing changes in corolla veins at different stages of closure. Scale bar = 0.5 cm. **(B–K)** Segregation of spiral vessels observed by light **(B–G)** and scanning electron microscopy **(H–K)**. The changes in the vessels at S1 and S7 are shown in **(B–D,H,I)** and **(E–G,J,K)**, respectively. Scale bars = 200 μm in **(B,E)**, 40 μm in **(C,D,F,G)**, 50 μm in **(H,J)**, and 30 μm in **(I,K)**.

We then investigated the morphological changes in the corolla veins during corolla closure (Figure 3A). At the full-bloom period, the acuminate veins were erect and smooth. The veins started to curve during S3 and further developed an undulating shape during S5. We further examined the morphological changes in the vessels of corolla veins. During the opening period (S1), all vessels were present in a vertical position (Figures 3B–D). Similar results were observed by SEM (Figures 3H,I). However, the vessels obviously curved during the complete closure stage (stage S7; Figures 3E–G,J,K).

### Transcriptome Analysis of Corolla Closure and Clustering of Differentially Expressed Genes

**FIGURE 4:**
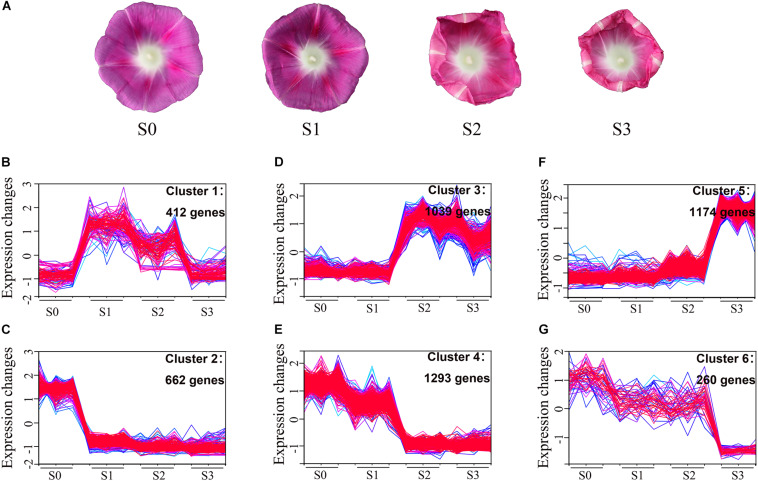
Analysis of differentially expressed genes in six clusters during corolla closure. **(A)** Photographs of samples collected at different time points used for transcriptome analysis. **(B–G)** An overview of the different clusters. The genes differentially expressed during corolla closure at different time points (S0, S1, S2, and S3) were grouped into 6 clusters, whose gene expression patterns are shown.

To examine the global transcription regulatory network in *I*. *purpurea* during corolla closure based on morphological features, we performed RNA-seq of samples from the upper corolla that were individually collected at S0, S1, S2, and S3 (Figure 4A). Samples selected from the same regions of the corolla during flower opening (S0) were used as controls. Sequencing of 12 independent libraries generated approximately 45.27 million raw tags per library. After the low-quality tags were filtered out, the total number of clean tags per library ranged from 41.96 to 43.48 million. In total, among the 120,462 unigenes identified, 71,146 (59.06%) unigenes were annotated against the NCBI non-redundant (NR) database using BlastX, and 52,936 coding sequences were obtained (Supplementary Table 1 and Supplementary Figure 3A). Principal component analysis (PCA) showed that the samples could be divided into four distinct groups: S0, S1, S2, and S3 (Supplementary Figure 3B). Differentially expressed genes (DEGs) were chosen based on a fold change of ≥ 4 [|log_2_ (fold change)| ≥ 2], *q*-value<0.05, and FPKM ≥ 1 in at least one stage. 4,840 DEGs were obtained for subsequent analysis (Supplementary Table 2).

Genes with similar expression patterns are often functionally correlated. To explore the dynamic changes in gene expression during corolla closure, we grouped the 4,840 DEGs into six clusters based on their expression pattern using *Mfuzz* software (Figures 4B–G and Supplementary Table 2). The cluster number and the corresponding expression values for each DEG in the clusters are shown in Figures 4B–G and Supplementary Table 2. The differentially expressed genes were placed into three major groups based on their expression patterns. Group I genes were differentially expressed from S0 to S1, including 412 upregulated genes in cluster 1 and 662 downregulated genes in clusters (Figures 4B,C). Group II genes showed major changes in expression between S1 and S2, and included 1039 upregulated genes in cluster 3 and 1,293 downregulated genes in cluster 4 (Figures 4D,E). Group III included genes with different expression patterns in S2 vs. S3, with 1,174 upregulated genes in cluster 5 and 260 downregulated in clusters 6 (Figures 4F,G). These results indicate that corolla closure is not a monotonic process; instead, the corolla passes through drastic transitions at specific points during the closure process.

### Analysis of DEGs Reveals the Chronology of Events During Corolla Closure

**FIGURE 5:**
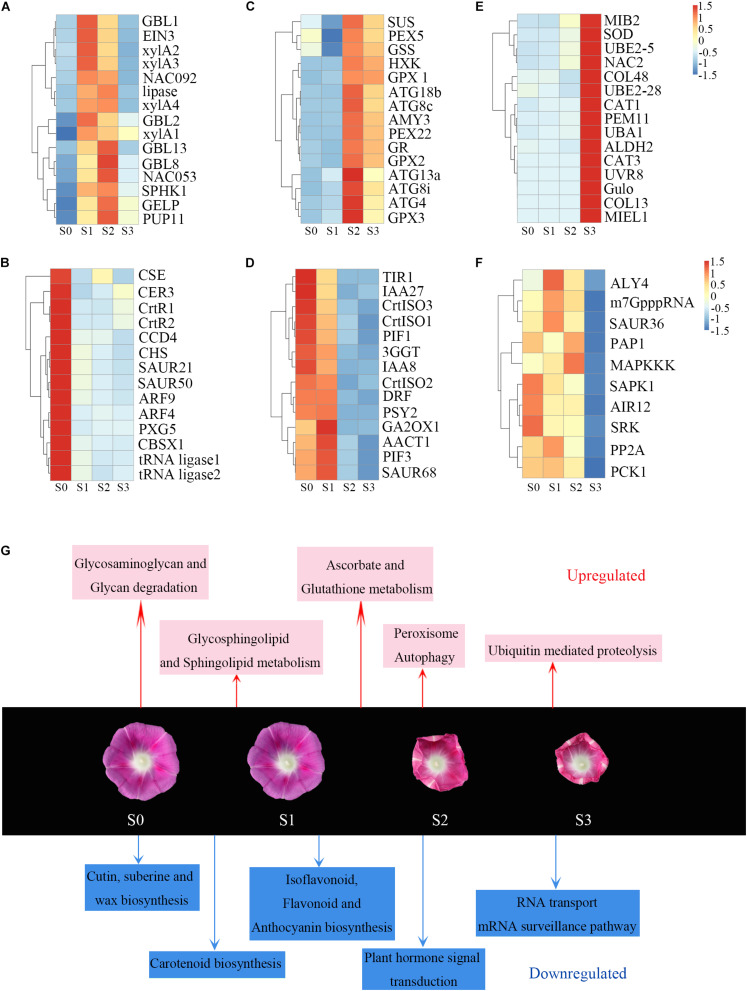
Heat maps of induced and repressed genes and metabolic processes during corolla closure. **(A–F)** Heat maps of upregulated and downregulated genes in different clusters. Abbreviations are described in Supplementary Table 3. **(G)** Metabolic processes induced and repressed at different time points during corolla closure. Red and blue indicate the induced and repressed processes, respectively.

The DEGs between S1 and S0 were shown in clusters 1 and 2. Genes in cluster 1 were immediately upregulated during this process. KEGG pathway analysis indicated that these genes were enriched in terms related to polysaccharide metabolism pathways including glycosaminoglycan and glycan degradation (Supplementary Figure 4A). The glycosphingolipid and sphingolipid metabolism pathways were also significantly enriched in this cluster (Supplementary Figure 4A). Plant cell walls are rigid, flexible structures composed of extensive layer of polysaccharides, proteins, and lignin. Also upregulated genes during corolla closure were 16 β*-galactosidase* (*GBL*) genes, which encode enzyme involved in glycosaminoglycan and glycan degradation (Figure 5A and Supplementary Table 2). This process, which is thought to release free galactosyl residues of galactolipids and glycoproteins (Smith and Gross, 2000; O’Donoghue et al., 2017), can severely degrade cell wall components to disrupt the integrity of the corolla. Next, we found that *Ethylene insensitive 3* (*EIN3*) and *NAC transcription factor* (*NAC092*, *NAC053*) were significantly overexpressed at S1 (Figure 5A). These three genes serve as senescence markers (Li et al., 2013; Zhang et al., 2019; Xiong et al., 2020; Cheng et al., 2021). Therefore, our pathway enrichment analysis indicated that senescence genes are involved in the early corolla closure process. Genes in cluster 2 had lower expression levels in S1 than in S0. The significantly enriched terms for these DEGs included cutin and wax biosynthesis, carotenoid biosynthesis, plant hormone signal transduction, and amino acid metabolism (Supplementary Figure 4B). The cutin and wax biosynthesis pathway genes, *Caffeoyl Shikimate Esterase* (*CSE*) and *Eceriferum 3* (*CER3*) were significantly downregulated (Figure 5B). CSE is an enzyme that plays a central role in lignin biosynthesis (Vanholme et al., 2013). Downregulation of this signaling pathway results in changes in cell wall synthesis and reduced lignin production (Zuk et al., 2016). CER3 is a biosynthetic enzyme involved in the production of cuticles of *Arabidopsis* leaves (Kim et al., 2019). The downregulation of genes in the cutin and cell wall biosynthesis pathways indicates that the biosynthesis of cell wall components is blocked in the closing corolla, which reduces the hardness and mechanical force of the corolla. The carotenoid metabolism genes β*-carotene hydroxylase* (*CrtR*) and *Carotenoid cleavage dioxygenase 4* (*CCD4*) were significantly downregulated in S1 vs. S0 (Figure 5B). These genes encode enzyme primarily responsible for the biosynthesis of zeaxanthin and xanthoxin, respectively (Taylor et al., 2005). In plant with blocked zeaxanthin and xanthoxin biosynthesis, abscisic acid (ABA) contents significantly decreased, resulting in more rapid water loss (Taylor et al., 2005; Du et al., 2010). Apparently, the activation of *GBL* and the suppression of *CSE* and *CER3* during S1, which are known to be involved in cell wall remodeling, highlight the important role of cell wall organization during corolla closure.

During S2, the corolla begins to curl inward; genes in clusters 3 and 4 are associated with this process. Genes in clusters 3 were significantly upregulated from S1 to S2, whereas genes in cluster 4 were downregulated during this process. Interestingly, the cellular processes of autophagy and antioxidant signaling were first observed during S2 (Supplementary Figure 4C). The expression of many autophagy-related (ATG) genes (*ATG4*, *ATG8c*, *ATG8i*, *ATG13a*, and *ATG18b*) involved in autophagy signaling rapidly increased from S1 to S2 (Figure 5C), indicating that an intracellular process occurs involving the vacuolar degradation of cytoplasmic components (Bassham et al., 2006). Also, *sucrose synthase* (*Sus*) and *glucose sensor hexokinase* (*HXK*) were significantly upregulated during stage S2. HXK is a sugar sensor that may act as a signaling molecule to induce senescence (Wingler et al., 2010; Dar et al., 2014; Kim, 2019). Glutathione-related genes in the antioxidant pathway, such as *glutathione synthetase* (*GSS*), *glutathione reductase* (*GR*) and *glutathione peroxidase* (*GPX*), were upregulated (Figure 5C) during this process; these changes might help maintain oxidative balance to protect cells against oxidative stress. In addition, *Cytokinin oxidase/dehydrogenase* (*CHK*), encoding an enzyme that irreversibly catalyzes the degradation of cytokinin (Werner et al., 2006), was also found in cluster 3 (Supplementary Table 2). These results indicate that the main process that occurs during this stage is not cell division but rather the activation of senescence. Unlike those in cluster 3, genes in cluster 4 were significantly downregulated from S1 to S2. Genes in this cluster were significantly enriched for growth-related pathways such as auxin as well as their receptors *Transport inhibitor response 1* (*TIR1*) and members of a number of gene families including *AUX/IAA* (*IAA8*, *IAA27*) and *SAUR* (*SAUR68*) involved in auxin signaling (Figure 5D). These changes indicate that corolla growth was severely inhibited during S2. Meanwhile, the lower expression levels of *Dihydroflavonol 4-reductase* (*DFR*), *prolycopene isomerase* (*CrtISO1*, *CrtISO3*) and *phytoene dehydrogenase* (*CarB*) at stage S2 (Figure 5D and Supplementary Table 2), which are crucial for carotenoid and anthocyanin formation, were similar to the gene expression patterns during senescence (Fang et al., 2008; Chai et al., 2011; Lou et al., 2014). Overall, these results suggest that sugar metabolism further activates autophagy to promote corolla closure.

At S3, the cells in the adaxial side of the corolla had collapsed and the upper part of the corolla was fully folded inward. A previous study also observed that the upper corolla begins to lose water and exhibits features of senescence during this process (Gui et al., 2016). To investigate the dynamic biological process from S2 to S3, we analyzed the expression of various signaling and regulatory genes during this period. The DEGs in cluster 5, which were significantly upregulated from S2 to S3 (Figure 5E), were mainly enriched in peroxisome and ubiquitin-mediated proteolysis. Peroxisomes metabolize reactive oxygen species (ROS). *Peroxisomal membrane protein 11* (*PEX11*), which were enriched in cluster 5, encode a protein required for peroxisome division (Frick, 2017). *Superoxide dismutase* (*SOD*) and two *catalase* (*CAT*) genes, encoding important antioxidant enzymes that scavenge ROS, were also expressed at higher levels at S3. The ubiquitin proteasome pathway is an efficient pathway for protein degradation that functions in plant hormone signaling, cell cycle regulation, and senescence in plants (Frugis and Chua, 2002). *Ubiquitin-activating enzyme E1* (*UBA1*), *ubiquitin-conjugating enzyme E2* (*UBE2-5*, *UBE2-28*), and *ubiquitin-protein ligating enzyme E3* (*MIB2*, *MIEL1*) were significantly upregulated from S2 to S3 (Figure 5E). By contrast, genes in cluster 6 were expressed at lower levels in S3 than S2 (Figure 5F). The enrichment of downregulated genes involved in RNA transport, the mRNA surveillance pathway, and the citrate cycle (TCA cycle) indicates that metabolic activity decreased during this stage. Above all, during S3, the corolla became physiologically dysfunctional, with signs of vacuole collapse, infiltration, and leakage, and peroxidase- and ubiquitin-mediated protein hydrolysis (Gui et al., 2016). These changes were accompanied by the downregulation of genes involved in RNA transport, mRNA surveillance pathway, and the citrate cycle (TCA cycle). These are obvious features associated with senescence during the last stage of corolla closure.

Overall, our results present a highly informative picture of the timeline of corolla closure and reveal specific transcriptional changes during this process (Figure 5G). The degradation of polysaccharides and the disturbance of cell wall synthesis prepare the cell wall for reorganization during S1. Many senescence-related transcription factors genes were regulated in S1; these transcription factors activate the downstream *ATG* genes and restrain carotenoid formation. Antioxidant, antioxidant enzyme, and peroxisome activities were enhanced to scavenge ROS and maintain redox equilibrium. During closure, the corolla entered into senescence and had reduced metabolic activity.

**FIGURE 6:**
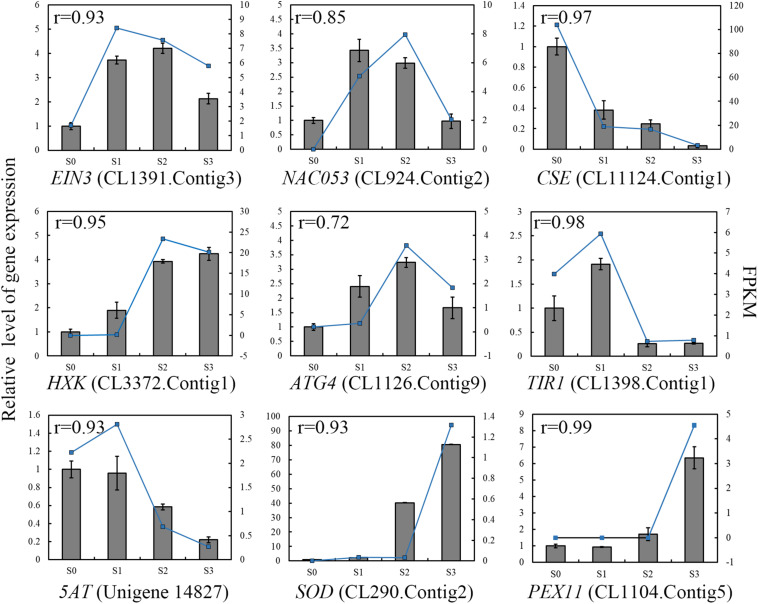
qRT-PCR validation of the levels of selected transcripts during corolla closure. The columns represent relative gene expression levels of independent biological replicates (left *y*-axis) determined by real-time qRT-PCR. The blue lines represent the expression levels of the transcripts (right *y*-axis). The correlation coefficient shown in the top left corner was calculated using R (3.5.3). EIN3, Ethylene Insensitive 3; NAC053, Transcription factor NAC domain-containing protein 53; CSE, Caffeoyl Shikimate Esterase; HXK, Hexokinase; ATG4, Autophagy-related protein 4; TIR1, Transport Inhibitor Response 1; 5AT, anthocyanin 5-aromatic acyltransferase; SOD, Superoxide Dismutase; PEX11, Peroxisomal Membrane Protein 11.

To validate the results obtained by RNA-seq, we performed qRT-PCR of selected genes. We used Pearson correlation analysis to assess the correlation between the different platforms. Overall, the qRT-PCR data closely agreed with the RNA-seq results (Figure 6). The gene expression patterns observed by both methods showed similar trends, further supporting the underlying molecular changes that occur during corolla closure.

## Discussion

### The Structures of Bulliform Cells and Acuminate Veins Jointly Contribute to Corolla Closure

Pollen germination significantly decreases at high ambient temperatures or when the corolla is removed (Liu et al., 2020). These observations imply that when the corolla adopts a bell shape during closure, it forms a micro-environment to ensure pollen germination and pollen tube growth (Liu et al., 2020). In the current study, morphological and anatomical analyses revealed the characteristics of corolla closure in *I. purpurea*. Based on these data, we propose that two specific structures, the adaxial bulliform cells and the acuminate veins, play central roles in corolla closure. During the early stage of blooming, the bulliform cells of the adaxial epidermis exhibited a papillate shape, with pigments on the corolla limb and intermediate parts of the corolla (Figure 1B and Supplementary Figure 2). Specifically, the bulliform cells can be distinguished from the smaller, irregular, flat cells on the abaxial surface. In contrast to the slight shrinkage of abaxial cells, the bulliform cells in the adaxial epidermis showed obvious breakage during the semi-closure period (Figure 1C). The destruction of bulliform cells is likely to result from different turgor pressure on both sides of the corolla, similar to the rolling up of corolla in *Ipomoea tricolor* due to a differential turgor between two sides of epidermis (Hanson and Kende, 1975). Indeed, the expansion and contraction of maize leaves are known to be regulated by the intracellular turgor pressure in bulliform cells (Matschi et al., 2020). Meanwhile, many genes involved in carbohydrate metabolism, such as *sucrose synthase*, and *glycosyl hydrolase*, were significantly induced during corolla closure (Figure 5). These genes encode enzyme that may facilitate the degradation of starch and sugar into small molecules such as fructose and glucose, which can contribute to changes in osmotic pressure (Wingler et al., 2010; Kim, 2019). Overall, these changes result in a lower water potential, which alters the intracellular turgor pressure, ultimately triggering the closure of the corolla.

Veins in the leaves and flowers of plants play essential roles in supplying water and maintaining the shapes of plant organs. Here we demonstrated that the veins of the corolla comprise spiral vessels that are acuminate and distributed in the shape of a pentagram (Figures 2F,G). Interestingly, in flowers with the incomplete corollas due to insect gnawing, the remaining acuminate veins caused the corolla to fold inward and finally close (Figure 2). When we artificially removed the acuminate veins, the corolla failed to close into a bell shape, indicating that the acuminate veins play an important role in corolla closure. Compared to the erect, smooth veins at the opening stage, the veins showed obvious curving at the closure stage (Figure 3), which affected the transport of water and accelerated corolla shrinkage. Similarly, the vein plays an essential role in corolla closure in *Ipomoea tricolor* (Phillips and Kende, 1980). Transcriptomic analysis revealed that *Caffeoyl Shikimate Esterase*, encoding an enzyme central to lignin biosynthetic (Vanholme et al., 2013), was downregulated in corollas at S1. The genes encoding β-glucosidase (i.e., cellobiase) and β-galactosidase (GBL), which are involved in cellulose degradation (Smith and Gross, 2000; O’Donoghue et al., 2017), were upregulated during S1. These results suggest that cell wall integrity is severely disrupted and likely contributes to the deformation of spiral vessels during corolla closure. Reduction in cell wall thickness, which is critical for inward fold of the veins during corolla closure, were found in another morning glory species *Ipomoea tricolor* (Phillips and Kende, 1980). Taken together, these findings suggest that the inward curving of the corolla is caused by the weakening of the mechanical support from veins, along with differences in turgor pressure on the two sides of the corolla.

### Corolla Closure Is Accompanied by Senescence

A previous study showed that cell wall material and organelles begin to degrade during early flower senescence, whereas, plastids, mitochondria, Golgi bodies, and endoplasmic reticulum are absorbed into vacuoles during flowering (Gui et al., 2016). Using SEM, we also determined that the epidermal cells in the adaxial side of the corolla shrank during corolla closure (Figure 1C), suggesting that corolla closure is closely related to cell senescence. A high-resolution, highly replicated time-course analysis of gene expression during corolla closure from the full-bloom stage (S0) to semi-closure (S3) revealed progressive changes in gene expression associated with the alterations in specific metabolic pathways. Genes upregulated during S1 after full bloom were enriched for carbohydrate metabolism (e.g., β*-galactosidase* and *UDP-glycosyltransferase 89*). These upregulated genes included *long-chain acyl-CoA synthetase 6* (*LACS6*), which encodes the enzyme that catalyzes the first step in fatty acid β-oxidation in the peroxisome to provide an energy source for senescence (Shockey et al., 2002). The glycosaminoglycan-degradation-related β*-galactosidase* (*GBL*), *purine permease* (*PUP11*), and *xylose isomerase* (*xylA1*, *xylA2*, *xylA3*, *xylA4*) were also upregulated during this process, which is similar to their expression pattern during leaf senescence in *Arabidopsis* (Breeze et al., 2011). Downregulated genes were significantly enriched for pathways involved in amino acid metabolism (such as tyrosine, arginine, and proline metabolism) and DNA replication. DNA fragmentation in the corolla is known to take place during the flowering stage (Gui et al., 2016). The downregulation of these groups of genes reflects the reduced cellular biosynthetic activity at S1. Finally, *Ethylene insensitive 3* (*EIN3*), encoding a key transcription factor involved in the ethylene response pathway that functions as a senescence marker gene (Li et al., 2013), was also induced during S1. The transcription factor genes *NAC092* and *NAC053* showed similar expression patterns to *EIN3* (Figure 5A), indicating that these transcription factors regulate the expression of many genes during senescence (Zhang et al., 2019).

Senescence is generally associated with massive nutrient remobilization and export from deteriorating organs (Rolland et al., 2006; Kim, 2019). In this study, the genes encoding α-amylase, β-glucosidase, and sucrose synthase, which are involved in starch and sucrose metabolism, were upregulated during corolla closure. These enzymes degrade starch and sucrose into glucose and fructose (Kim, 2019). Glucose and fructose accumulate in senescent leaves, indicating that carbohydrates can act as regulators of autophagy (Izumi and Ishida, 2011). Autophagy is an intracellular process that involves the vacuolar degradation of cytoplasmic components (Yoshimoto et al., 2004). The genes involved in autophagy (*ATG4*, *ATG8*, *ATG13*) and senescence (*senescence-specific gene 39*, *SAG39*) were upregulated at S2 (Figure 5). These results are consistent with the ultrastructural observations in *Ipomoea* during early senescence with pronounced changes in the volume of empty vacuoles and the degradation of organelles (Phillips and Kende, 1980; Gui et al., 2016). Finally, *Hexokinase* (*HXK*), encoding a core component of plant sugar sensing and signaling (Rolland et al., 2006), was highly expressed during corolla closure. The correlation between *HXK* expression and the rate of senescence pointed to the important role of HXK-dependent sugar signaling in leaf senescence (Rolland et al., 2006). These results suggest that starch and sucrose contents decrease during corolla closure, which is similar to senescence.

In summary, in this study, we characterized the cellular basis and transcriptome profiles of *I*. *purpurea* during corolla closure and showed that metabolic changes induce cellular shrinkage associated with corolla closure. At the opening stage (from S0 to S1), many genes enriched in the polysaccharide degradation pathway and genes encoding positive regulators of cell wall reorganization are upregulated. Senescence-related transcription factors genes also emerge at this stage. At S2 (i.e., the early senescence stage), the corolla edge curls inward, and starch and sucrose are degraded into glucose and fructose, leading to changes in cellular turgor pressure. The larger bulliform cells of the adaxial epidermis shrink prior to cells on the abaxial side, and downstream autophagy- and senescence-related genes are activated. Finally, RNA transport, transcription regulator activity, and the TCA cycle are enriched among genes downregulated during S3, indicating that cellular biosynthetic activity shuts down during corolla closure. Our findings will greatly facilitate the modeling of corolla closure in the future.

## Data Availability Statement

The datasets presented in this study can be found in online repositories. The names of the repository/repositories and accession number(s) can be found below: National Genomics Data Center (NGDC) under the BioProject accession number PRJCA004887 (https://ngdc.cncb.ac.cn/).

## Author Contributions

PZ and JL conceived the experiments. PZ, MS, XW, MG, and YS performed the experiments. PZ, YS, RG, and LZ analyzed the data. PZ, LZ, and JL wrote and revised the manuscript. LZ and TW critically edited the manuscript. All authors approved the final manuscript.

## Conflict of Interest

The authors declare that the research was conducted in the absence of any commercial or financial relationships that could be construed as a potential conflict of interest.

## Publisher’s Note

All claims expressed in this article are solely those of the authors and do not necessarily represent those of their affiliated organizations, or those of the publisher, the editors and the reviewers. Any product that may be evaluated in this article, or claim that may be made by its manufacturer, is not guaranteed or endorsed by the publisher.

## Footnotes

1
https://ngdc.cncb.ac.cn/

2
http://www.genome.jp/kegg/

## Supplementary Material

Supplementary Figure 1 The corolla determines flower closure movements. **(A)** Closure of a corolla without stamens or a pistil. **(B–D)** Photographs of corolla closure in flowers lacking 1/3, 2/3, or all of the colorful corolla. Scale bar = 2 mm.

Supplementary Figure 2 Transverse sections from different parts of a corolla generated by freehand dissection. **(A)** Corolla limb, **(B)** intermediate, and **(C)** Corolla tube parts of corolla. Scale bars = 40 μm.

Supplementary Figure 3 Annotation and principal component analysis (PCA) of transcriptomic changes in different stages of corolla closure. **(A)** Pie chart showing the species distribution. **(B)** PCA of transcriptomes at stages S0 to S3. The circles around the points represent different stages.

Supplementary Figure 4 Significantly enriched KEGG pathways in each cluster. Circle size represents the enrichment score and colors (from green to red) represent the significance of enrichment (from low to high) calculated by hypergeometric test. **(A)** cluster 1; **(B)** cluster 2; **(C)** cluster 3; **(D)** cluster 4; **(E)** cluster 5; **(F)** cluster 6.

Supplementary Table 1 List of unigenes quality indices of the 12 samples.

Supplementary Table 2 List of differentially expressed genes during corolla closure.

Supplementary Table 3 List abbreviation of DEGs in each heat map.

Supplementary Table 4 List of primers used for qRT-PCR analysis.

Supplementary Movie 1 The process of corolla closure in *I*. *purpurea*. The sequence of corolla closure was filmed by time-lapse photography using a camera programmed to take a photograph every minute. The entire process (until full corolla closure) took 4.5 h.
