# Supplementary figures and images for: Morphological Characterization and Transcriptional Regulation of Corolla Closure in Ipomoea purpurea
Source: Front Plant Sci. 2021 Sep 7;12:697764. doi: 10.3389/fpls.2021.697764 (PMC8453026; doi:10.3389/fpls.2021.697764)

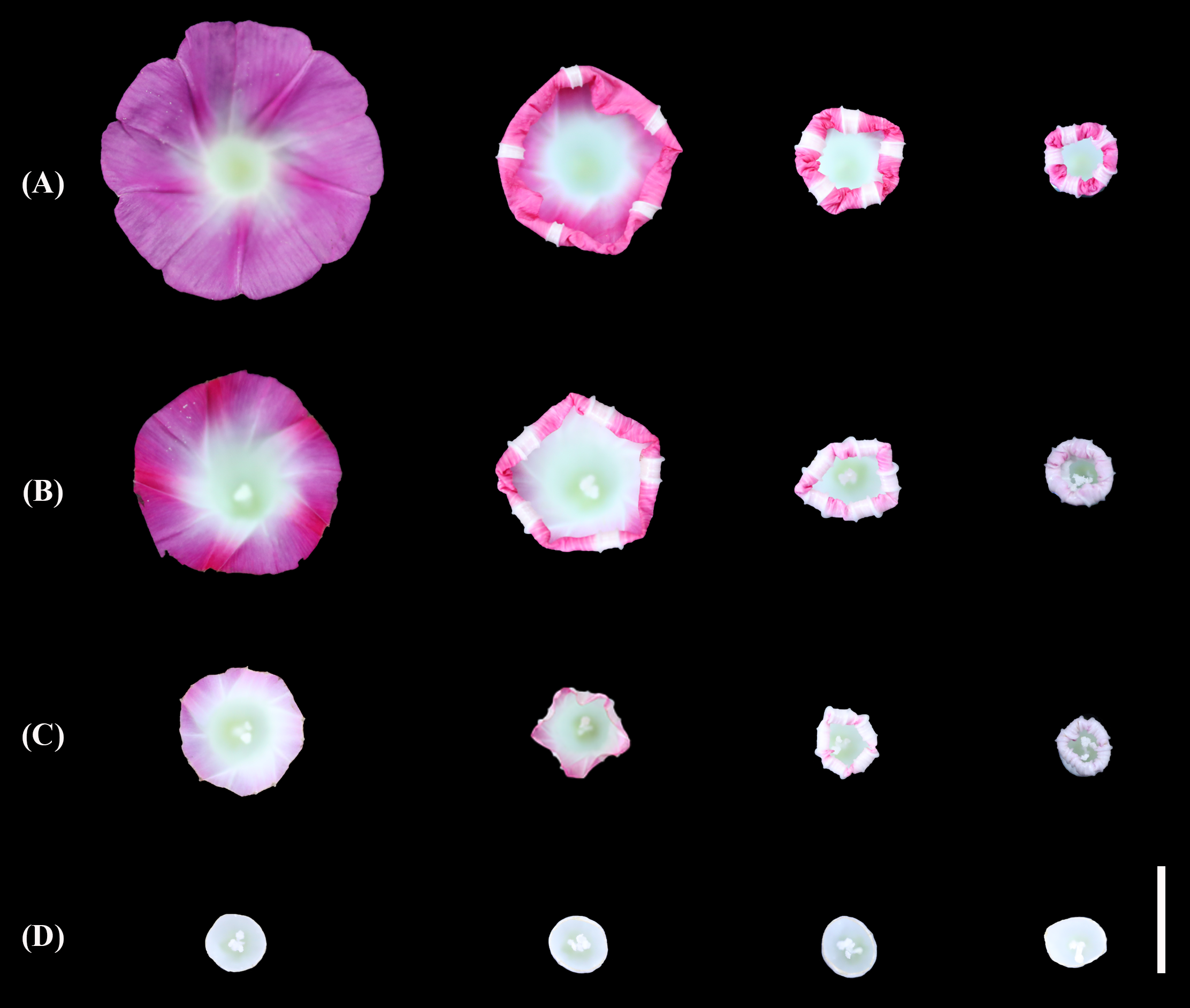

Supplement: Supplementary Figure 1 — The corolla determines flower closure movements. (A) Closure of a corolla without stamens or a pistil. (B–D) Photographs of corolla closure in flowers lacking 1/3, 2/3, or all of the colorful corolla. Scale bar = 2 mm. [file Image_1.TIF]

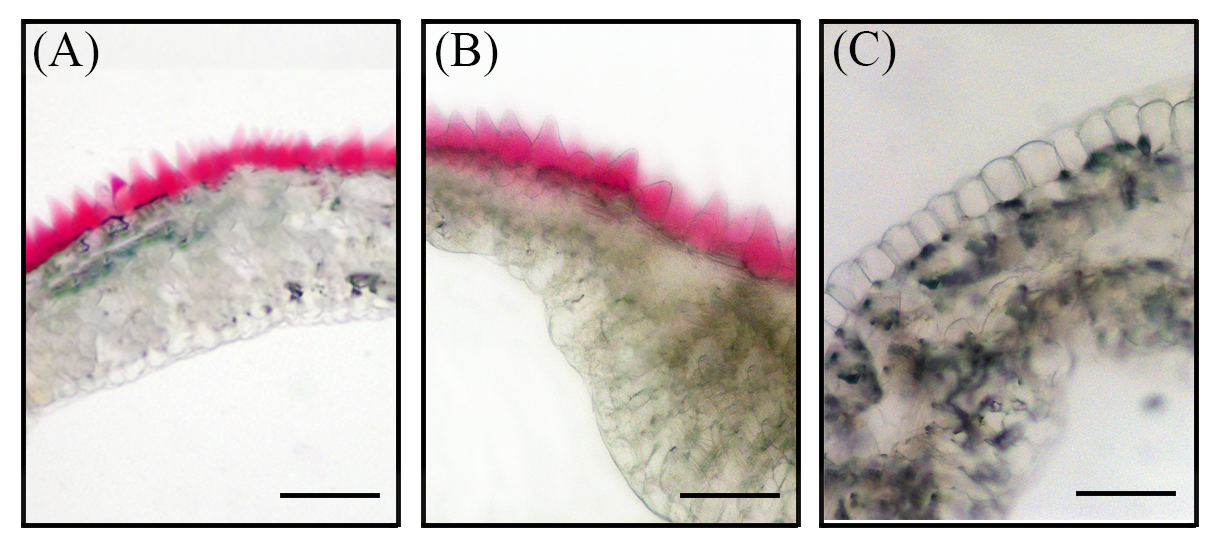

Supplement: Supplementary Figure 2 — Transverse sections from different parts of a corolla generated by freehand dissection. (A) Corolla limb, (B) intermediate, and (C) Corolla tube parts of corolla. Scale bars = 40 μm. [file Image_2.TIF]

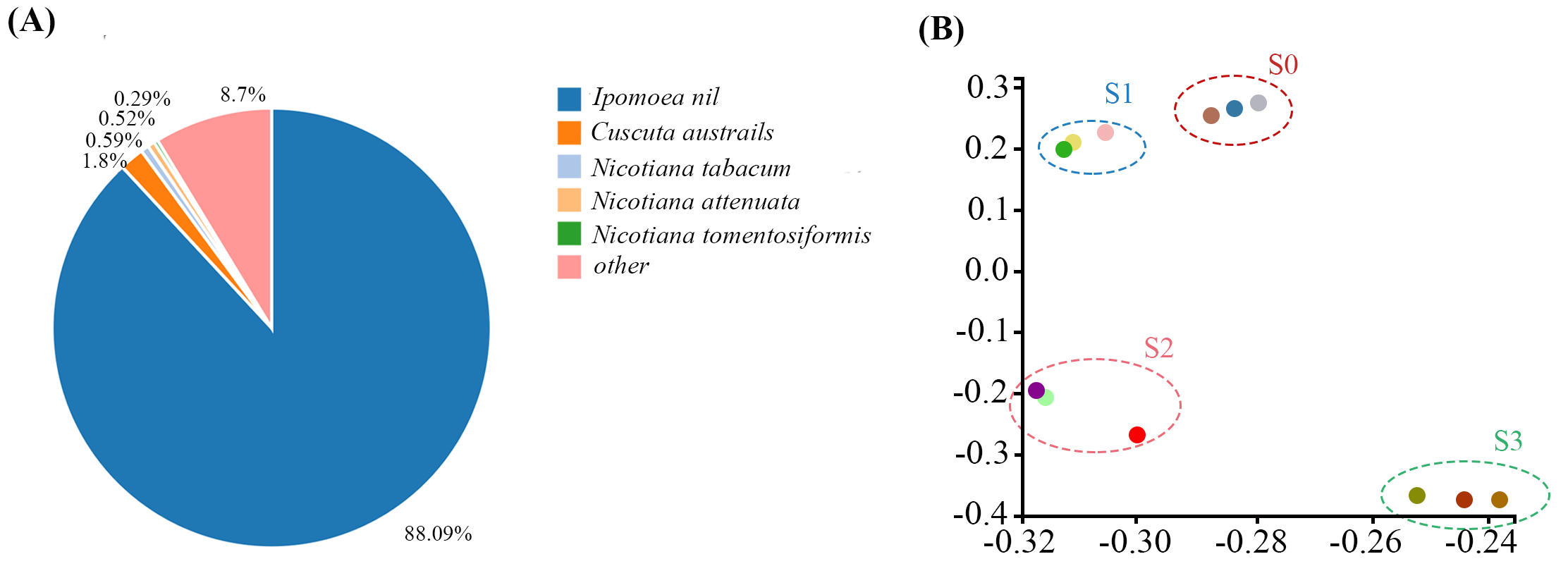

Supplement: Supplementary Figure 3 — Annotation and principal component analysis (PCA) of transcriptomic changes in different stages of corolla closure. (A) Pie chart showing the species distribution. (B) PCA of transcriptomes at stages S0 to S3. The circles around the points represent different stages. [file Image_3.TIF]

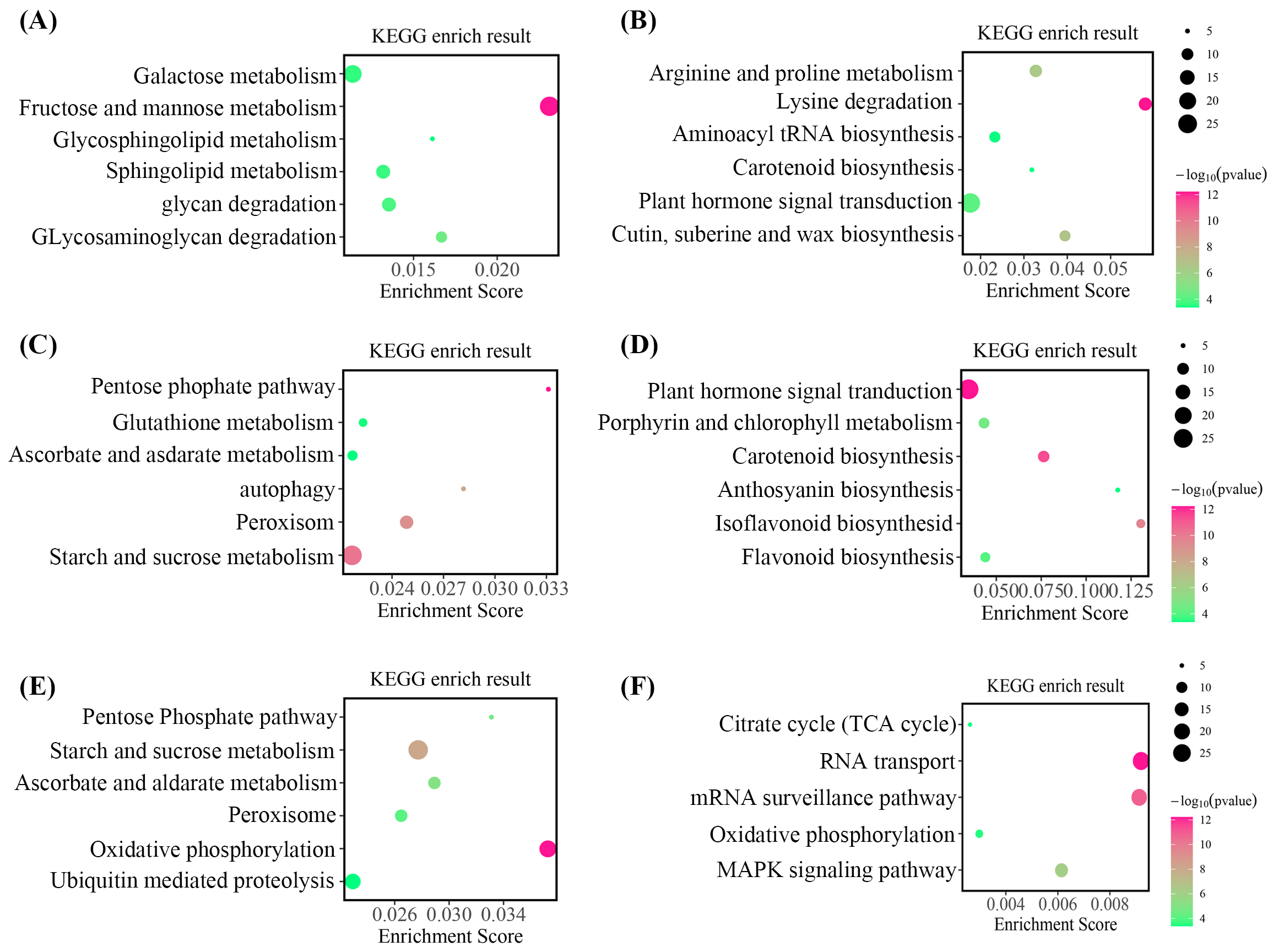

Supplement: Supplementary Figure 4 — Significantly enriched KEGG pathways in each cluster. Circle size represents the enrichment score and colors (from green to red) represent the significance of enrichment (from low to high) calculated by hypergeometric test. (A) cluster 1; (B) cluster 2; (C) cluster 3; (D) cluster 4; (E) cluster 5; (F) cluster 6. [file Image_4.TIF]
